# Socioeconomic factors and use of psychotherapy in common mental disorders predisposing to disability pension

**DOI:** 10.1186/s12913-022-08389-1

**Published:** 2022-08-01

**Authors:** Helena Leppänen, Olli Kampman, Reija Autio, Tino Karolaakso, Turkka Näppilä, Päivi Rissanen, Sami Pirkola

**Affiliations:** 1 Faculty of Medicine and Health Technology, Tampere University and Tampere University Hospital, Tampere, Finland; 2 Pirkkala Municipal Health Centre, Pirkkala, Finland; 3 Department of Psychiatry, Tampere University Hospital, Pirkanmaa Hospital District, Tampere, Finland; 4 Faculty of Social Sciences (Unit of Health Sciences), Tampere University, Tampere, Finland; 5 Faculty of Social Sciences (Psychology), Tampere University, Tampere, Finland; 6 Tampere University Library, Tampere University, Tampere, Finland

**Keywords:** Psychotherapy, Common mental disorder, Depression, Anxiety, Disability pension, Socioeconomic status

## Abstract

**Background:**

Research in high-income countries has identified low socioeconomic status as a risk factor for disability pension (DP) due to common mental disorders (CMDs). Psychotherapy is an evidence-based treatment for the majority of CMDs along with medication and it is often targeted to prevent work disability. This study examines socioeconomic differences in the use of rehabilitative psychotherapy in Finland, where citizens have universal health coverage, but psychotherapy is partly dependent on personal finance.

**Methods:**

The study subjects (*N =* 22,501) were all the Finnish citizens granted a DP due to CMD between 2010 and 2015 and a comparison group (*N =* 57,732) matched based on age, gender, and hospital district. Socioeconomic differences in psychotherapy use were studied using logistic regression models. Socioeconomic status was defined by education, income, and occupation. Age, gender, and family status were also examined.

**Results:**

A lower level of education, lower occupational status (blue-collar worker), male gender, and older age, were associated with less frequent psychotherapy use, in both groups. Education was the strongest component of socioeconomic status associated with psychotherapy use, but the role of income was not straightforward. Unemployment when approaching DP, but not otherwise, was a risk factor for not receiving rehabilitative psychotherapy. Socioeconomic disparities were not any smaller among CMD patients approaching DP than in the comparison group.

**Conclusion:**

This study demonstrates the disparity in the provision of psychotherapy for CMD patients, even on the verge of DP with an acute need for services. This disparity is partly related to a complex interplay of socioeconomic factors and the service system characteristics. Factors predisposing to unequal access to mental health services are presumably diverse and should be studied further.

**Supplementary Information:**

The online version contains supplementary material available at 10.1186/s12913-022-08389-1.

## Introduction

Disability to work due to mental disorders is growing, and increasing costs due to health care expenditure and lost working years are a concern in high-income countries (HICs) [1, 2]. Common mental disorders (CMDs) including depressive and anxiety disorders [3] are highly prevalent globally [4] and major causes of disability pensions (DPs) [5]. The World Health Organization’s (WHO) estimate of the proportion of the global population with depression is 4.4% and with anxiety 3.6% [3]. In Finland, mental disorders are the most common reason for DPs (53%, 103,000 persons in 2019), and depression is the most common mental disorder [6].

Socioeconomic status (SES) is often defined by the factors: income, education, and occupation, to reflect a person’s social, material, and psychological resources [7, 8]. Low SES is associated with poorer mental health [7, 9] and higher work disability due to mental disorders [8, 10]. People with lower SES, especially lower education, tend to use mental health services less willingly than people with higher SES [11–13], even though the need may be higher [7, 9].

Psychotherapy is an evidence-based treatment for depression and anxiety disorders [14, 15] and is recommended solely or in combination with pharmacotherapy [16, 17]. Recent literature indicates that there is an association between SES and psychotherapy use, and it varies according to the service system, as shown in British [18] and French [19] survey studies and a German [20] register data study. The British survey study [18] found that higher levels of education, income, and occupational status were all associated with more frequent use of private psychotherapy, when payment by the client was 40–100 GBP per visit. However, the British system offered also public psychotherapy which was used more frequently by people with lower income, education, and occupational status, even though public psychotherapy was not as readily available, due to a need for a referral and longer waiting times before receiving treatment. The French survey study [19] examined the role of life events, mental health status, and sociodemographic characteristics, including education, in determining psychotherapy use. The study included mainly middle-class, employed people with specific profession-based health insurance. Education was positively associated with psychotherapy use in different severities of psychiatric disorders, despite the fact that the study population was well-informed and with high-quality insurance cover [19]. In the German register study [20] of employed people with statutory health insurance, the positive associations of education and occupation with psychotherapy use were evident, whereas the association of income was rather weak, which was presumably attributable to the absence of financial barriers to outpatient psychotherapy in Germany. On the other hand, income-based inequities in psychotherapy use have been shown in Canada and Australia [21]. In both countries, the unmet need for psychotherapy was higher at low-income levels, even though the service systems differ. Canada has a two-tier system causing often higher psychotherapy costs for low-income people, whereas Australia has provided universally affordable psychotherapy since Better Access reform in 2006. The results indicated that income-based inequities in psychotherapy existed also in Australia, despite the reform. This was explained by an anti-stigma initiative in 2000 and better access to psychotherapy, when both demand and unmet need were supposedly increased especially among low-income Australians during the study period.

In Finland, rehabilitative psychotherapy may be partially compensated by the Social Insurance Institution (SII) to people whose ability to study or work is impaired due to a mental disorder, who have received at least 3 months of appropriate therapy, and to whom rehabilitative psychotherapy is considered necessary (based on a psychiatrist’s statement) to improve or support the ability to work or study [22, 23]. In addition to the SII compensation, which in 2020 was 57.60 euros per session [23], clients pay 30–70 euros per session out of their own pockets, depending on the therapist’s prices. Financial support for psychotherapy is available for up to 80 sessions per year and for up to a total of 200 sessions in 3 years [22]. The number of people using psychotherapy supported by the SII has grown from less than 10,000 in 2005 to over 50,000 in 2019 [24]. One notable reason for this increase is that rehabilitative psychotherapy was changed from being discretionary to mandatory in 2011, hence making its use no longer dependent on the SII budget, but on the need of the applicant [25]. Although the supply of psychotherapy was made less restricted, a shortage of psychotherapists remains, and it may take several months for a patient to find a psychotherapist. In addition to psychotherapy compensated by the SII, there is some psychotherapy for which patients pay entirely out of their own pockets, or in rarer cases is paid for by hospital districts as medical rehabilitation for patients, not within the scope of the services provided by the SII [22].

The present register study is part of the RETIRE research project, which studies the risk factors for DPs granted on grounds of mental health [26]. We aim to examine the socioeconomic equality of the Finnish service system in targeting psychotherapy at CMD patients prior to a temporary or permanent DP, and their comparison group. We hypothesise that psychotherapy use is lower in low SES groups, mostly due to financial barriers. Furthermore, we hypothesise that socioeconomic disparities in psychotherapy use are smaller among CMD patients approaching DP (CMD + DP group) than in the comparison group, because people in higher SES have more probably seeked for rehabilitative psychotherapy already at earlier stages of their mental health condition due to better-resourced occupational health services and higher mental health literacy [12, 22]. Furthermore, in the CMD + DP group, the need for rehabilitative psychotherapy is relatively urgent, and the health care system has to offer all the feasible treatment and rehabilitation measures, before the justification of DP. In addition, sociodemographic factors, age, gender, and family type are studied because of their assumed importance in explaining mental health [4, 27, 28] and help-seeking [11]. The aim of the present study is to complement the current research literature on the role of SES factors in psychotherapy use, in the context of a Nordic welfare state.

## Methods

### Study population

We obtained data from the registers of the SII, the Finnish Centre for Pensions, the National Institute for Health and Welfare, and Statistics Finland. The data combined medical, socioeconomic, and sociodemographic information on the study subjects. A previous RETIRE publication [8] described the formation of the original dataset including all mental disorders. We used a subset of the original data, and our dataset included all Finnish citizens granted a temporary or permanent DP for the first time between 2010 and 2015 due to CMD (ICD-10: F32–F34 depressive disorders and F40–43 anxiety, stress-related, and adjustment disorders, *N =* 22,501) [29]. CMD diagnoses are listed with ICD-coding in Table 1. Diagnoses were recorded from the decisions granting DPs and the main diagnosis leading to DP was used. A comparison group was matched for gender, age, and hospital district from the population registers of Statistics Finland (*N =* 57,732), and it included only people, who were not awarded a DP due to mental disorders in 2010 - 2015. The matching was done in a year DP was granted for the people in the CMD + DP group. Possible diagnoses of the people in the comparison group were not recorded, because they lacked a DP grant or respective document providing this information. People with previous pensions were excluded from the dataset, but people in the comparison group may have been granted DP due to any other than mental health condition within the follow-up of 3 years after the DP. The comparison group was used to detect whether the possible socioeconomic differences in psychotherapy use are smoother among CMD patients approaching DP than among other people, hence reflecting the ability of the service system to provide services more efficiently for those in urgent need, regardless of SES.

**Table 1:** Principal CMD diagnosis and use of rehabilitative psychotherapy in CMD + DP group

CMD diagnosis	Frequency (%) *n =* 22,501	Psychotherapy use total = 4000
F32 Major depressive disorder	9632 (42.8)	1313 (13.6)
F33 Major depression, recurrent	9935 (44.2)	2077 (20.9)
F34 Cyclothymia/persistent mood disorder	496 (2.2)	86 (17.3)
F40 Phobic anxiety disorder	505 (2.2)	102 (20.2)
F41 Other anxiety disorder	1138 (5.1)	235 (20.7)
F42 Obsessive-compulsive disorder	356 (1.6)	115 (32.3)
F43 Reaction to severe stress, and adjustment disorders	439 (2.0)	72 (16.4)

*Abbreviations*: *CMD* Common mental disorder, *DP* Disability pension

DP granted in 2010-2015

### Socioeconomic and sociodemographic factors

Following the literature, the three factors selected to represent various dimensions of SES were: education, income, and occupational status [9]. Sociodemographic factors sex, age, and family type were also measured. The values used for those in the CMD + DP group were taken from 1 year before going on DP, and for those in the comparison group, the respective year was used. Age was measured in the year when DP was granted, and again the respective year was used for the comparison group.

Education was categorized into five groups in line with the classification of Statistics Finland. Basic level refers to a maximum of 9 years of education, which is the length of compulsory comprehensive school in Finland. Upper secondary education means spending 11 to 12 years in basic education, including upper secondary education or vocational school. Short-cycle tertiary education lasts from two to 3 years after upper secondary education and includes qualifications that are not polytechnic degrees. Lower degree tertiary education entails three to 4 years of education after upper secondary education and comprises polytechnic degrees and lower university degrees. Higher degree tertiary education comprises education lasting at least 5 to 6 years after upper secondary education leading to masters’ degrees, licentiates, or doctoral degrees.

Income (including salaries, entrepreneurial income, capital income, transfer payments and fringe payments) was calculated for each person by dividing the income of the person’s household (measured by Statistics Finland) with the OECD consumption unit, indicating the sum of the weights of its members [30]. The resulting average income in euros per person per year was divided into quintiles based on the data: lowest (less than 14,454 euros), middle-lower (14,455e – 20,468e), middle (20,469e – 25,931e), middle-higher (25,932e – 33,254e) and highest (more than 33,255e).

Occupational status was classified into seven groups following the classification of Statistics Finland: blue-collar worker, lower white-collar worker, upper white-collar worker, entrepreneur, agriculture and forestry entrepreneur, student, and unemployed.

Of sociodemographic factors, gender included males and females, age was categorized into five groups, and family type was categorized into four groups: living alone; couple (living together with a partner without children); couple with children (living with a partner and one or more children); and single parent.

### Rehabilitative psychotherapy

Information on psychotherapy was retrieved from the statistics of the Social Insurance Institution, which makes the decisions, based on a psychiatrist’s statement, on who is eligible to be granted rehabilitative psychotherapy. Eligibility is determined by impaired studying or working ability due to a mental disorder, after receiving at least 3 months of appropriate treatment after the diagnosis. The information was collected in the 9-year interval, which was 5 years prior to the year when DP was granted, the granting year, and 3 years after. We considered this as the most important period to rehabilitate CMD patients in order to prevent or postpone permanent work disability. In the Finnish disability retirement system, a person in most cases first spends approximately 1 year (300 working days) on sickness benefit, and often a temporary disability pension is granted before a permanent one, with some anticipation of the individual being able to return to work. For the comparison group, the study period was the matching 9-year interval, but with no connection to any specific life event. Psychotherapy was considered realized if therapy visits numbered more than zero within the 9-year study period.

### Statistical analysis

Logistic regression was used to detect the associations between the different exposures, i.e. socioeconomic and sociodemographic factors, and the realization of psychotherapy during the 9-year interval. The reference group used in the analysis, in age was the highest, and in income and education the lowest category. A grand mean was used for variables family status and occupation, because these variables were not ordinal and hence not that easily interpreted with a reference group. Crude models, meaning univariate models were created for each exposure separately. As a final model, a multivariable logistic regression model was created where these factors were entered simultaneously, and the model was adjusted for all factors. The odds ratios (OR) and confidence intervals (CIs) were computed. To test statistical significance, a very small *p*-value < 0.001, and accordingly, 99.9% CIs were used due to the large sample size. Some SES information was missing regarding income and occupation. The missing values were omitted from the regression analysis as shown in Table 2. The collinearity of the exposures was assessed with generalized variance inflation factor (GVIF) adjusted for each exposure based on the degrees of freedom. Since all exposures resulted in a GVIF below two, there was no indication of issues with collinearity. Statistical analyses were conducted with SPSS Statistics Version 25 (IBM Inc., Armonk NY), and R version 4.0.1 with package ggplot2 version 3.3.1 and car version 3.0.

**Table 2:** Socioeconomic and sociodemographic characteristics and use of rehabilitative psychotherapy in CMD + DP group and comparison group

	CMD + DP group		Comparison group	
	Psychotherapy recipients (%)	Total	Psychotherapy recipients (%)	Total
**Total**	**4000 (17.8)**	**22,501**	**844 (1,5)**	**57,732**
Gender				
Male	957 (11.3)	8452	116 (0.5)	21,442
Female	3043 (21.7)	14,049	728 (2.0)	36,290
Age				
18–25 years	600 (29.1)	2065	111 (2.3)	4807
26–35 years	871 (31.5)	2767	204 (2.8)	7189
36–45 years	818 (22.4)	3656	203 (2.0)	10,174
46–55 years	1125 (16.2)	6932	235 (1.2)	18,837
56–65 years	586 (8.3)	7081	91 (0.5)	16,725
Family status				
Living alone	1306 (15.7)	8344	250 (2.1)	12,143
Couple	1103 (16.0)	6907	199 (1.0)	19,510
Single parent	486 (21.1)	2305	115 (2.7)	4255
Couple with children	1105 (22.3)	4945	280 (1.3)	21,824
Education				
Basic level	531 (9.2)	5765	62 (0.7)	9401
Upper secondary level	1694 (16.2)	10,462	284 (1.1)	26,260
Short cycle tertiary	679 (21.4)	3176	129 (1.4)	9311
Lower degree tertiary	537 (34.2)	1572	144 (2.3)	6171
Higher degree tertiary	559 (36.6)	1526	225 (3.4)	6589
Income ª				
Lowest	785 (13.2)	5935	141 (2.1)	6624
Middle-lower	831 (16.5)	5022	134 (1.4)	9361
Middle	741 (17.6)	4215	164 (1.4)	11,978
Middle-higher	769 (21.2)	3624	198 (1.4)	13,726
Highest	839 (24.9)	3370	200 (1.3)	15,497
Occupation ^b^				
Agriculture and forestry entrepreneur	32 (13.1)	244	4 (0.3)	1159
Entrepreneur	192 (16.6)	1157	45 (1.1)	4263
Upper white-collar worker	758 (30.2)	2511	239 (2.4)	10,146
Lower white-collar worker	1356 (21.7)	6261	303 (1.6)	19,349
Blue-collar worker	380 (10.1)	3763	60 (0.5)	12,932
Student	638 (33.1)	1929	104 (4.1)	2525
Unemployed	191 (8.5)	2256	67 (1.2)	5726

*Abbreviations*: *CMD* Common mental disorder, *DP* Disability pension

^a^ CMD + DP group total *N =* 22,166; Comparison group total *N =* 57,186

^b^ CMD + DP group total *N =* 18,121; Comparison group total *N =* 56,100

### Ethical issues

The study was based on register data collected regularly for administrative, development, and evaluation purposes. No patients were contacted individually, and none will be recognizable from the data. The Ethics Committee of the National Institute of Health and Welfare approved the plan of the project.

## Results

### Descriptive analysis

The mean age in the CMD + DP group was 46.7 years (SD 12.4 years), and gender representation was 37.6% males. Age and gender distributions of the matched comparison group were similar. Minor differences were due to the exclusion criteria of the study subjects specified in the previous RETIRE publication [8]. The most common CMDs leading to DP were major depressive disorder (F32*) and recurrent depression (F33*), whereas anxiety disorders were far less common as principal diagnoses. The distribution of psychotherapy use between different CMD diagnoses is presented in Table 1.

**Fig. 1:**
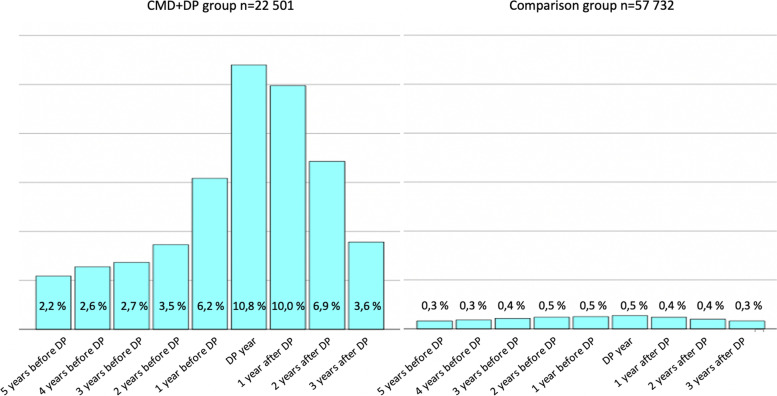
Distribution of psychotherapy years around DP year in CMD + DP group and comparison group

Socioeconomic and sociodemographic characteristics and psychotherapy use in the CMD + DP group and the comparison group are presented in Table 2. In the CMD + DP group, altogether 4000 (17.8%) people attended psychotherapy in the 9-year period around the granted DP. In the comparison group, 844 (1.5%) people attended psychotherapy in the corresponding 9-year interval. Because the comparison group includes also people without CMDs, the psychotherapy use in that group is certainly lower than in the CMD + DP group, and the rates themselves are not comparable. Diagnoses in the comparison group were not known, but the prevalence of CMDs was supposed to follow the general estimates, being 5,6% for depressive and 3,2% for anxiety disorders in Finland [3], and psychotherapy is in over 90% of cases received due to CMDs [31]. Psychotherapy years were distributed around the DP year as shown in Fig. 1. To be noted, each person may receive psychotherapy 1-3 years and hence it can be distributed in 1-4 calendar years, depending on the timing of psychotherapy sessions. In the comparison group, the psychotherapy use was quite evenly distributed in the study period. In the CMD + DP group, the peak number of psychotherapy years was in the DP year.

### Association of socioeconomic and sociodemographic factors with psychotherapy use

**Fig. 2:**
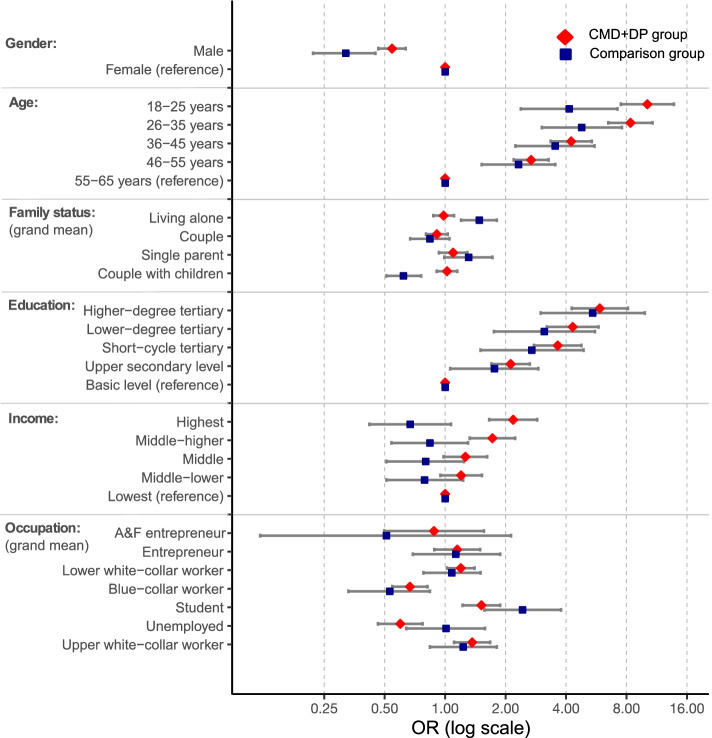
Socioeconomic and sociodemographic factors associated with psychotherapy use separately for CMD + DP group and comparison group

ORs and CIs for psychotherapy use by different socioeconomic and sociodemographic factors are presented in Table 3. The crude model studied these factors separately, and the final model was adjusted for all factors. The ORs of the final model are likewise presented in Fig. 2. The crude and final models differed from each other to a minor extent, and we display the results of the final models below.

**Table 3:** Socioeconomic and sociodemographic factors associated with psychotherapy use separately for CMD + DP group and comparison group

	CMD + DP group (*n =* 22,501)				Comparison group (*n =* 57,732)			
	Crude model		Final model		Crude model		Final model	
	OR	99.9% CI	OR	99.9% CI	OR	99.9% CI	OR	99.9% CI
Gender								
Male	0.46 *	0.41 – 0.53	0.54 *	0.47 – 0.64	0.27 *	0.19 – 0.37	0.32 *	0.22 – 0.45
Female (reference)	1.00		1.00		1.00		1.00	
Age								
18–25 years	4.54 *	3.67 – 5.62	10.17 *	7.50 – 13.78	4.32 *	2.70 – 6.90	4.15 *	2.38 – 7.21
26–35 years	5.09 *	4.19 – 6.19	8.38 *	6.50 – 10.80	5.34 *	3.52 – 8.10	4.79 *	3.03 – 7.59
36–45 years	3.20 *	2.63 – 3.87	4.25 *	3.35 – 5.37	3.72 *	2.45 – 5.65	3.53 *	2.24 – 5.55
46–55 years	2.15 *	1.80 – 2.57	2.68 *	2.20 – 3.27	2.31 *	1.54 – 3.47	2.32 *	1.52 – 3.54
56–65 years (reference)	1.00		1.00		1.00		1.00	
Family status (deviation)								
Living alone	0.81 *	0.74 – 0.89	0.98	0.87 – 1.11	1.26 *	1.04 – 1.52	1.48 *	1.20 – 1.81
Couple	0.83 *	0.76 – 0.92	0.91	0.80 – 1.03	0.62 *	0.50 – 0.76	0.84	0.67 – 1.05
Single parent	1.17 *	1.02 – 1.34	1.10	0.93 – 1.29	1.66 *	1.29 – 2.14	1.31	0.99 – 1.72
Couple with children	1.26 *	1.14 – 1.40	1.02	0.91 – 1.15	0.78 *	0.65 – 0.94	0.62 *	0.51 - 0.76
Education								
Higher degree tertiary	5.70 *	4.53 – 7.17	5.89 *	4.27 – 8.13	5.33 *	3.31 – 8.56	5.43 *	2.99 – 9.87
Lower degree tertiary	5.11 *	4.06 – 6.44	4.32 *	3.21 – 5.81	3.60 *	2.18 – 5.95	3.12 *	1.75 – 5.57
Short cycle tertiary	2.68 *	2.18 – 3.30	3.64 *	2.77 – 4.78	2.12 *	1.27 – 3.53	2.70 *	1.50 – 4.89
Upper secondary level	1.90 *	1.60 – 2.27	2.12 *	1.70 – 2.64	1.65 *	1.04 – 2.62	1.76 *	1.06 – 2.91
Basic level (reference)	1.00				1.00		1.00	
Income								
Highest	2.18 *	1.81 – 2.61	2.18 *	1.66 – 2.87	0.60 *	0.42 – 0.87	0.67	0.42 – 1.07
Middle-higher	1.77 *	1.47 – 2.12	1.72 *	1.33 – 2.23	0.67 *	0.47 – 0.97	0.84	0.54 – 1.30
Middle	1.40 *	1.17 – 1.68	1.26	0.98 – 1.62	0.64 *	0.44 – 0.94	0.80	0.51 – 1.24
Middle-lower	1.30 *	1.09 – 1.55	1.20	0.95 – 1.52	0.67 *	0.45 – 1.00	0.79	0.51 – 1.23
Lowest (reference)	1.00		1.00		1.00		1.00	
Occupation (deviation)								
Agriculture and forestry entrepreneur	0.72	0.42 – 1.29	0.88	0.50 – 1.56	0.29	0.07 – 1.20	0.51	0.12 – 2.13
Entrepreneur	0.94	0.74 – 1.21	1.15	0.88 – 1.50	0.90	0.55 – 1.47	1.13	0.69 – 1.88
Upper white-collar worker	2.05 *	1.74 – 2.42	1.36 *	1.11 – 1.68	2.03 *	1.47 – 2.80	1.23	0.84 – 1.81
Lower white-collar worker	1.31 *	1.14 – 1.51	1.20 *	1.02 – 1.40	1.34	0.98 – 1.83	1.08	0.78 – 1.50
Blue-collar worker	0.53 *	0.44 – 0.64	0.67 *	0.55 – 0.82	0.39 *	0.25 – 0.61	0.53 *	0.33 – 0.84
Student	2.35 *	1.97 – 2.79	1.52 *	1.22 – 1.88	3.61 *	2.46 – 5.31	2.43 *	1.57 – 3.78
Unemployed	0.44 *	0.35 – 0.56	0.60 *	0.46 – 0.77	1.00	0.65 – 1.54	1.01	0.64 – 1.58

Socioeconomic and sociodemographic differences in psychotherapy use, odds ratio (OR) and 99.9% confidence interval (99.9% CI), separately for common mental disorder + disability pension (CMD + DP) group and comparison group matched for gender, age and hospital district

Crude model: Logistic regression model for all factors separately

Final model: Multivariable logistic regression model adjusted for all factors in the table

Reference categories: Gender: female, Age: 56-65 years, Education: basic level, Income: lowest

Family Status and Occupation were analysed as a deviation from the grand mean

Statistical significance * *p <* 0.001

Male gender was decidedly associated with lower psychotherapy use in both groups; in the CMD + DP group: OR 0.54 (99.9% CI 0.47–0.64) and the comparison group: OR 0.32 (99.9% CI 0.22–0.45). Age was negatively associated with psychotherapy use in both groups. In the CMD + DP group, OR for the youngest age group was 10.17 (99.9% CI 7.50–13.78), and OR declined towards the oldest age group, which was the reference. In the comparison group, this negative trend was smoother but still perceptible. Family status was only significant in the comparison group. Living alone was associated with higher psychotherapy use (OR 1.48; 99.9% CI 1.20–1.81) than average (meaning the grand mean of all family statuses), and couples with children with lower psychotherapy use (OR 0.62; 99.9% CI 0.51–0.76) than average.

Education was positively associated with psychotherapy use. For those with the highest level of education in the CMD + DP group, OR was 5.89 (99.9% CI 4.27–8.13) and in the comparison group, OR was 5.43 (99.9% CI 2.99–9.87) when the reference was the lowest education. Income level was associated with psychotherapy use only in the CMD + DP group, and only for the two highest income groups: OR 2.18 (99.9% CI 1.66–2.87) and OR 1.72 (99.9% CI 1.33–2.23) respectively. Occupation was associated with psychotherapy use in the CMD + DP group; white-collar workers and students had positive, whereas blue-collar workers and unemployed people had negative associations. In the comparison group, the respective associations were seen only in students (positive) and blue-collar workers (negative).

Socioeconomic and sociodemographic differences in psychotherapy use, by odds ratios (OR) and 99.9% confidence intervals (CI) of final statistical models, adjusted for all factors in the figure, separately for common mental disorder + disability pension (CMD + DP) group (diamond) and comparison group (square). Reference groups for each factor marked as 1.0, except family status and occupation were analysed as a deviation from the grand mean.

SES was measured 1 year before DP, whereas the use of psychotherapy was measured in the 9-year interval around DP. This creates a potential risk of reverse causation because in some cases SES was measured only after the person had already received psychotherapy. Changes in occupational status, as an estimate of SES, in the 4-year interval, from 5 years to 1 year before DP, are shown in Supplementary Tables 1 and 2. Occupational status is dynamic and has changed in both groups. Most notably, in the comparison group, the number of students has decreased in the follow-up of 4 years, whereas the number of students in the CMD + DP group has increased. Logistic regression of an association of occupational status 5 years before DP was run to evaluate the risk of reverse causation. This was the first year of the study period for each person, and the first possible year to receive psychotherapy. The results are shown in Supplementary Table 3. Similar trends in associations between SES and psychotherapy use were detected than in the original analysis. Hence, our data showed no evidence of reverse causation.

## Discussion

We found considerable socioeconomic differences in psychotherapy use in our comprehensive national-level data. We studied the common mental disorder (CMD) patients on the verge of disability pension (DP), when, at the latest, rehabilitation services should be in maximal use. Lower socioeconomic status (SES) was associated negatively with psychotherapy use according to all factors: income, education, and occupation. To the best of our knowledge, this was the first study, which specifically focused on people on the verge of DP and included all SES groups, regardless of their insurance or employment status. In addition to our primary hypothesis, older age and male gender were negatively associated with psychotherapy use. Our study findings corroborate those reported in earlier research [18–20] in other HICs. Unlike we hypothesized, socioeconomic disparities in psychotherapy use were not any smaller in the CMD + DP group than in the comparison group, which implies that disparities are present even with an urgent need for services.

Education showed a positive association with psychotherapy use in both groups, which is in line with earlier research [18–20, 32]. One explanation is that among people with lower levels of education, mental health literacy is lower and illness stigma is higher [12], which makes acceptance and help-seeking more complicated [33, 34]. Finding a therapist requires time and effort, which may be compromised among people with low SES, due to low MHL [12] and constrained living and working conditions [34]. Considering occupation, a pronounced gap in psychotherapy use among blue-collar workers may be linked to lower MHL due to lower levels of education [12] which hampers help-seeking and reduces willingness to spend money on psychotherapy. Furthermore, alcohol use disorder is among the most prevalent mental disorders among working-aged Finnish people, and blue-collar workers are at higher risk [7]. This co-morbidity may also prevent the treatment of CMDs.

Unemployed people attended psychotherapy notably less than the average in the CMD + DP group, but not in the comparison group. Unemployed includes both the temporary and the long-term unemployed. In Finland, two-thirds of long-term unemployed have health conditions that would render them eligible for a DP, half of them due to depression [35]. In our study, unemployed people with prolonged CMDs may have received rehabilitative interventions years before, outside the timeframe of our study, or they have become marginalized from services. Health services are provided in public, private, and occupational health care in Finland. Unemployed people have no access to occupational health care, which is usually free of charge for medical consultations and can often provide more preventive and early mental health interventions than municipal primary health care [22]. Personal factors like poor awareness of one’s own condition and feeling stigma may prevent help-seeking by unemployed people [36]. Also system-induced factors like miscommunication with professionals [36], financial barriers and lack of timely interventions may hinder unemployed people from obtaining the psychotherapy they need. In a previous RETIRE study we found no clear association for the regional number of psychotherapy recipients with regional all mental disorder DP risk [37]. This may indicate that psychotherapy is not successfully focused on people in the highest DP risk, which this study supports.

Income was positively associated with psychotherapy use in the highest and middle-higher (> 25,931 e) income categories among the CMD patients approaching DP when the reference was the lowest category. No corresponding association was found in the comparison group, presumably because people with lower SES have a higher prevalence of CMDs [7, 9] and correspondingly higher need for psychotherapy. Income-based unequal access to psychotherapy has also been reported in the UK [18] regarding private but not publicly provided psychotherapy, in Germany [20] to a minor extent because of the absence of financial barriers, and in Canada [21] with a two-tier system, in which psychological services are mostly to be paid for or are employment-based benefits, whereas the costs of medical consultations are fully covered by public health insurance, which resembles the Finnish system. In Finland, financial barriers to psychotherapy exist, despite partial reimbursement by the SII [22]. Therefore, we expected income to have a higher association with psychotherapy use than it had. Instead, education was one of the three interlinked SES factors most evidently associated with psychotherapy use.

This study also revealed sociodemographic inequalities in psychotherapy use. Older age was associated with less frequent psychotherapy use. This is in line with earlier research [38], even though the effect of psychotherapy for depression is not known to be any lower in older age groups [39]. Psychotherapy is offered well for young age groups and students, but many lost working years could be saved with more extensive rehabilitation for older working-age people with CMDs.

After adjustments, family status was associated with psychotherapy use only in the comparison group; people living alone attended more psychotherapy than did those living with a partner or children. CMD prevalence is higher among people living alone, which is partly explained by lack of social support, unemployment, and alcohol consumption [28]. Differences in CMD prevalence most probably explain the result in the comparison group.

Our study revealed a considerable gender difference; males attended psychotherapy decidedly less than females, which is in line with the literature [19, 32, 40]. The difference was greater in the comparison group, which is partly explained by the higher prevalence of depression among females [41], thus creating a greater need for psychotherapy. Excessive alcohol consumption especially among Finnish males [42] may inhibit recognition of CMDs, as well as help-seeking and rehabilitation for them. Males’ reluctance to seek help for mental health concerns has also been explained by cultural expectations, structural barriers, and unappealing service environments [40, 43]. Increasing awareness of mental health issues and reducing stigma in general could redefine acceptable ways to treat mental illnesses and lower the threshold for help-seeking.

### Societal implications

Finland is a Nordic welfare state with comprehensive social security, including health care and rehabilitation services for all residents. Psychotherapy is an evidence-based clinical practice for the treatment of depression and anxiety disorders [14–17], but psychotherapy is only partly compensated by social insurance in Finland and is not easily obtainable due to a shortage of psychotherapists. The CMD burden falls more heavily on people with low SES [9] but they attend psychotherapy less than do people with higher SES. The reasons for this are presumably diverse.

We suspect that the service system accentuates the socioeconomic gradient of the CMD burden by the unequal provision of services. Lower SES groups, as well as older working-aged people and males, should be empowered to attend psychotherapy more extensively, or if not appropriate, alternative treatment or rehabilitation should be offered. Timely intervention, psychoeducation and concrete guidance in finding a therapist should be provided, and payments out of people’s own pockets reduced to bridge the socioeconomic gap. A special effort to provide services for unemployed people should be made. Furthermore, more extensive provision of short-term psychotherapies free-of-charge or at low cost would most probably reduce the need for long psychotherapies, thereby reducing public healthcare expenditure in the long run. The National Mental Health Strategy 2020–2030 in Finland [44] recognizes the need to strengthen the basic level services, but concrete actions are still called for.

### Strengths, limitations and future research

The strength of this study is the use of comprehensive, high-quality national-level register data [45]. To the best of our knowledge, this is the first study with a unique research setting, which enabled us to study psychotherapy use by people granted a DP due to CMDs, with no exclusion of any SES factor categories. One limitation is that the data was lacking information on possible diagnoses of people in the comparison group because this information was not documented without a DP or other respective document. The idea behind the use of a comparison group didn’t suffer from this deficiency. However, a higher prevalence of CMDs, and hence higher demand for psychotherapy, among people with lower SES [9] had to be considered to affect the results by reducing socio-economic differences in the comparison group. Also, another limitation is that SES was measured 1 year before DP, and psychotherapy was received either before or after this. To evaluate the risk of reverse causation, we performed additional analysis and used occupational status 5 years before DP as an estimate of SES (i.e. the first year of the study period for each person). We detected similar trends in associations between SES and psychotherapy use (data shown in the supplement). Hence, our data showed no evidence of reverse causation. We used the WHO definition of CMDs [3], but according to some definitions, CMDs may also include substance abuse and impulse control disorders [4]. Furthermore, one limitation is that psychotherapy use was reported as a binary outcome, but the duration of psychotherapy (normally 1–3 years) or dropouts were not studied. Further research is needed to study socioeconomic equality in comprehensive CMD treatment, including medication, occupational rehabilitation, and psychotherapy.

## Conclusion

We found evidence of a complex interplay of socioeconomic and sociodemographic factors contributing to differences in obtaining access to reimbursed psychotherapy. The reasons for this disparity are presumably diverse. System-based factors widening the socioeconomic disparities in mental health merit further study.

## Supplementary Information

**Additional file 1: Supplementary Table 1.** Crosstabs of psychotherapy 5 years and 1 year before DP in the CMD+DP group. **Supplementary Table 2.** Crosstabs of psychotherapy 5 years and 1 year before DP in the comparison group. **Supplementary Table 3.** Associations between occupational status and psychotherapy use in CMD+DP group and comparison group.

## Data Availability

Register data were obtained from the registers of the Social Insurance Institution of Finland, The Finnish Centre for Pensions, THL, and Statistics Finland. The data were combined and stored in the server of Statistics Finland. Restrictions apply to the availability of these data, which were used under license for the current study, and so are not publicly available. More information on the data is available on reasonable request from the corresponding author.

## Abbreviations

CI: Confidence interval
CMD: Common mental disorder
DP: Disability Pension
GVIF: Generalized variance inflation factor
HIC: High income country
OR: Odds ratio
SES: Socioeconomic status
SII: Social Insurance Institution
WHO: World Health Organization
