# Supplementary material for: Socioeconomic factors and use of psychotherapy in common mental disorders predisposing to disability pension
Source: BMC Health Serv Res. 2022 Aug 1;22:983. doi: 10.1186/s12913-022-08389-1 (PMC9344663; doi:10.1186/s12913-022-08389-1)
Supplement: Supplementary file 1 — Additional file 1: Supplementary Table 1. Crosstabs of psychotherapy 5 years and 1 year before DP in the CMD+DP group. Supplementary Table 2. Crosstabs of psychotherapy 5 years and 1 year before DP in the comparison group. Supplementary Table 3. Associations between occupational status and psychotherapy use in CMD+DP group and comparison group. [file 12913_2022_8389_MOESM1_ESM.docx]

SUPPLEMENTARY DATA

Supplementary Table 1: Crosstabs of psychotherapy 5 years and 1 year before DP in the CMD+DP group

|  |  | Occupational status 1 year before DP | | | | | | | |  |
| --- | --- | --- | --- | --- | --- | --- | --- | --- | --- | --- |
|  |  | Agriculture and forestry entrepreneur | Entrepreneur | Upper white-collar worker | Lower white-collar worker | Blue-collar worker | Student | Unemployed | **Total** |  |
|  |  |  |  |  |  |  |  |  |  |  |
|  |  |  |  |  |  |  |  |  |  |  |
| Occupational status 5 years before DP | Agriculture and forestry entrepreneur | 161 (61.7) | 55 (21.1) | 3 (1.1) | 6 (2.3) | 15 (5.7) | 13 (5.0) | 8 (3.1) | **261** |  |
|  | Entrepreneur | 24 (2.2) | 647 (62.8) | 66 (6.2) | 108 (10.1) | 84 (7.8) | 46 (4.3) | 71 (6.6) | **1073** |  |
|  |  |  |  |  |  |  |  |  |  |  |
|  | Upper white-collar worker | 3 (0.1) | 79 (3.1) | 1811 (70.6) | 336 (13.1) | 69 (2.7) | 148 (5.8) | 119 (4.6) | **2565** |  |
|  |  |  |  |  |  |  |  |  |  |  |
|  | Lower white-collar worker | 10 (0.2) | 104 (1.8) | 280 (4.8) | 4553 (78.1) | 304 (5.2) | 269 (4.6) | 310 (5.3) | **5830** |  |
|  | Blue-collar worker | 20 (0.5) | 87 (2.3) | 91 (2.4) | 468 (12.3) | 2516 (65.9) | 271 (7.1) | 366 (9.6) | **3819** |  |
|  | Student | 10 (0.6) | 33 (2.2) | 104 (6.4) | 303 (18.6) | 215 (13.2) | 779 (47.7) | 189 (11.6) | **1633** |  |
|  | Unemployed | 13 (0.6) | 62 (3.1) | 100 (4.9) | 340 (16.8) | 414 (20.4) | 213 (10.5) | 884 (43.6) | **2026** |  |
|  | **Total** | **241 (1.4)** | **1094 (6.4)** | **2455 (14.3)** | **6114 (35.5)** | **3617 (21.0)** | **1739 (10.1)** | **1947 (11.3)** | **17 207*** |  |

*Altogether 5294 missing values due to age (<16 years), military service, or missing information in either of the variables.

Supplementary Table 2: Crosstabs of psychotherapy 5 years and 1 year before DP in the comparison group

|  |  | Occupational status 1 year before DP | | | | | | | |  |
| --- | --- | --- | --- | --- | --- | --- | --- | --- | --- | --- |
|  |  | Agriculture and forestry entrepreneur | Entrepreneur | Upper white-collar worker | Lower white-collar worker | Blue-collar worker | Student | Unemployed | **Total** |  |
|  |  |  |  |  |  |  |  |  |  |  |
|  |  |  |  |  |  |  |  |  |  |  |
| Occupational status 5 years before DP | Agriculture and forestry entrepreneur | 975 (81.3) | 64 (5.3) | 14 (1.2) | 42 (3.5) | 59 (4.9) | 29 (2.4) | 16 (1.3) | **1199** |  |
|  | Entrepreneur | 68 (1.8) | 2969 (77.6) | 113 (3.0) | 236 (6.2) | 208 (5.4) | 81 (2.1) | 150 (3.9) | **3825** |  |
|  |  |  |  |  |  |  |  |  |  |  |
|  | Upper white-collar worker | 12 (0.1) | 274 (2.7) | 7594 (75.8) | 1278 (12.8) | 206 (2.1) | 290 (2.9) | 367 (3.7) | **10 021** |  |
|  |  |  |  |  |  |  |  |  |  |  |
|  | Lower white-collar worker | 24 (0.1) | 310 (1.7) | 1498 (8.2) | 14144 (77.2) | 950 (5.2) | 457 (2.5) | 931 (5.1) | **18 314** |  |
|  | Blue-collar worker | 42 (0.3) | 359 (2.6) | 253 (1.9) | 1593 (11.7) | 9424 (69.1) | 487 (3.6) | 1490 (10.9) | **13 648** |  |
|  | Student | 7 (0.2) | 81 (2.2) | 355 (9.5) | 1065 (28.6) | 877 (23.5) | 816 (21.9) | 524 (14.1) | **3725** |  |
|  | Unemployed | 14 (0.4) | 110 (2.8) | 180 (4.6) | 648 (16.6) | 838 (21.4) | 214 (5.5) | 1904 (48.7) | **3908** |  |
|  | **Total** | **1142 (2.1)** | **4167 (7.6)** | **10 007 (18.3)** | **19 006 (34.8)** | **12 562 (23.0)** | **2374 (4.3)** | **5382 (9.8)** | **54 640*** |  |

*Altogether 3092 missing values due to age (<16 years), military service, or missing information in either of the variables.

Supplementary Table 3: Associations between occupational status and psychotherapy use in CMD+DP group and comparison group

|  |  | CMD + DP group | | Comparison group | |
| --- | --- | --- | --- | --- | --- |
|  |  | OR | 99.9 % CI | OR | 99.9 % CI |
| Occupational status  5 years before DP  (deviation) | Agriculture and forestry entrepreneur | 0.66 | 0.38 – 1.13 | 0.47 | 0.16 – 1.38 |
|  | Entrepreneur | 0.86 | 0.68 – 1.10 | 0.69 | 0.40 – 1.17 |
|  | Upper white-collar worker | 2.15 * | 1.84 – 2.52 | 1.82 * | 1.36 – 2.44 |
|  | Lower white-collar worker | 1.38 * | 1.21 – 1.59 | 1.25 | 0.95 – 1.65 |
|  | Blue-collar worker | 0.57 * | 0.48 – 0.68 | 0.49 * | 0.34 – 0.71 |
|  | Student | 2.02 * | 1.71 – 2.40 | 2.84 * | 2.04 - 3.96 |
|  | Unemployed | 0.51 * | 0.42 – 0.62 | 0.98 | 0.62 – 1.54 |

Occupational differences in psychotherapy use, analysed as a deviation from the grand mean, odds ratio (OR), and 99.9% confidence interval (99.9 % CI), separately for common mental disorder + disability pension (CMD+DP) group and comparison group matched for gender, age, and hospital district

Statistical significance * p<0.001
